# ^18^F-FDG Uptake Characteristics in Differentiating Benign from Malignant Nasopharyngeal Lesions in Children

**DOI:** 10.1155/2015/354970

**Published:** 2015-10-20

**Authors:** Chao Ma, Renjian Zou, Yanlei Huo, Suyun Chen, Shaoyan Wang, Shuqi Wu, Zhiyi Ye, Zhenyu Wu, Feng Fang, Hui Wang

**Affiliations:** Nuclear Medicine, Xinhua Hospital, Shanghai Jiao Tong University School of Medicine, Shanghai 200092, China

## Abstract

The characteristics of FDG uptake in the physiologic and malignant nasopharynx were investigated in the paper which was correlated with either pathologic findings or clinical follow-up. Three patients had pathologically established nasopharyngeal malignancy. In the 3 nasopharyngeal malignancies, 2 had diffusely and expansively increased FDG uptake, and one had asymmetric uptake. Our results indicated that the difference between adenoid hypertrophy and malignancy is asymmetric or diffusely expansive ^18^F-FDG uptake with or without correlating morphologic lesion on diagnostic CT in children under 10 years of age. The typical characteristics of physiologic and inflammatory ^18^F-FDG uptake in nasopharynx are symmetrically trapezoid. Diffusely increased nasopharyngeal FDG uptake can be considered physiologic if SUV_max_ is less than 7.6 but should be carefully assessed by pharyngorhinoscopy if SUV_max_ is greater than 11 and there is no correlating morphologic lesion on diagnostic CT. The diffusely, expansively increased uptake, and asymmetric uptake in particular, should be considered as malignancy. Further biopsy is especially indicated in patients with retropharyngeal space and bilateral cervical lymph node abnormality but no history of malignancy.

## 1. Introduction

Pediatric nasopharyngeal carcinoma (NPC) is rare and usually poorly differentiated [1]. ^18^F-FDG PET/CT is a valuable imaging modality for evaluating and monitoring NPC in children [2] and adults [3–6]. Asymmetric ^18^F-FDG uptake in the nasopharynx on PET with a correlating morphologic lesion on fully diagnostic CT may well indicate nasopharyngeal malignancy. However, diffusely increased ^18^F-FDG uptake in the pediatric nasopharynx is common for both physiologic and inflammatory changes. Therefore, it is difficult to tell whether diffusely increased ^18^F-FDG uptake in the nasopharynx on PET without a correlating morphologic lesion on diagnostic CT suggests nasopharyngeal malignancy. No studies of the probability of NPC vis-à-vis ^18^F-FDG uptake have, to our knowledge, been performed. In this investigation, we studied the utility of nasopharyngeal ^18^F-FDG uptake in children under 10 years of age to determine the characteristics of FDG uptake in the physiologic and malignant nasopharynx.

## 2. Materials and Methods

### 2.1. Study Population

From May 2011 to May 2013, 154 children under 10 years of age with either established or suspected malignancy were enrolled in the study and underwent ^18^F-FDG PET scanning. The study was approved by the research ethics committee of our hospital. Written informed consent was obtained from the parents or guardians of all included patients before ^18^F-FDG PET/CT. The distribution of malignancy and suspected malignancy is listed in Table 1.

### 2.2. ^18^F-FDG PET/CT Imaging Protocol

^18^F-fluorine was produced at the PET Centre of Xinhua Hospital, Shanghai. Blood glucose level was measured prior to injection of ^18^F-FDG and was normal in all. The patients received a 5.18 MBq/kg dose of ^18^F-FDG (±10%; maximum, 444 MBq) intravenously after an overnight fast or, for afternoon studies, a minimally 4-hour fast. After injection, the patients stayed in the PET preparation room and rested for 1 hour. Relaxation with no movement or minimal movement during the uptake phase was encouraged. Just before the end of the 1-hour uptake period, the patients voided their bladders.

Transmission CT images for attenuation correction and lesion localization, and PET emission images, were acquired approximately 1 hour after injection using a Biograph PET/CT system (Siemens). The CT images were acquired using slice thickness of 0.3 cm, 0.8 s tube rotation, table speed of 1.5 cm/rotation, pitch of 1.5 : 1, 120 kV, 90 mA, and dose modulation. The PET images were obtained from the vertex of the skull to the mid-thigh level for 5 min per bed position in 2-dimensional mode. The PET/CT scans for each patient were reviewed by 2 nuclear medicine physicians without knowledge of any clinical information. SUV_max_ in the nasopharynx was determined from manually placed regions of interest over the area of tumor activity in multiple planes. Follow-up clinical and pathologic information were recorded for correlation with tumor uptake of ^18^F-FDG (SUV_max_).

### 2.3. Statistical Analysis

We analyzed the association between SUV_max_ in the nasopharynx and clinical and pathologic results using the 50th, 75th, 90th, and 95th percentiles for differentiation between benign and malignant nasopharyngeal lesions in children (SPSS software, version 13.0; IBM).

## 3. Results

The children had a median age of 4 years, and the male-to-female ratio was 91 : 63. One hundred thirty-seven had established malignancy and underwent PET/CT for staging, postoperative restaging, or therapeutic assessment. The other 17 underwent PET/CT because of suspected malignancy or fever of unknown origin.

**Figure 1:**
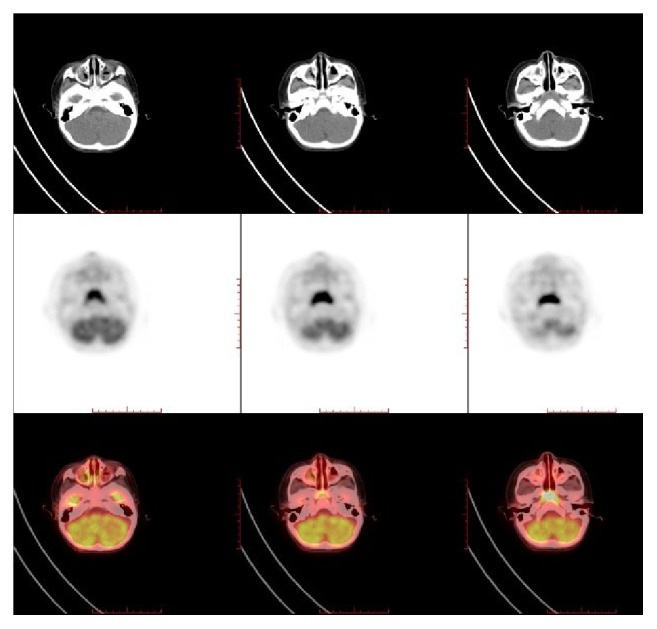
The normally increased uptake of ^18^F-FDG (SUV_max_ 11) in the nasopharynx of a 1-year-old boy is symmetric and trapezoid.

**Figure 2:**
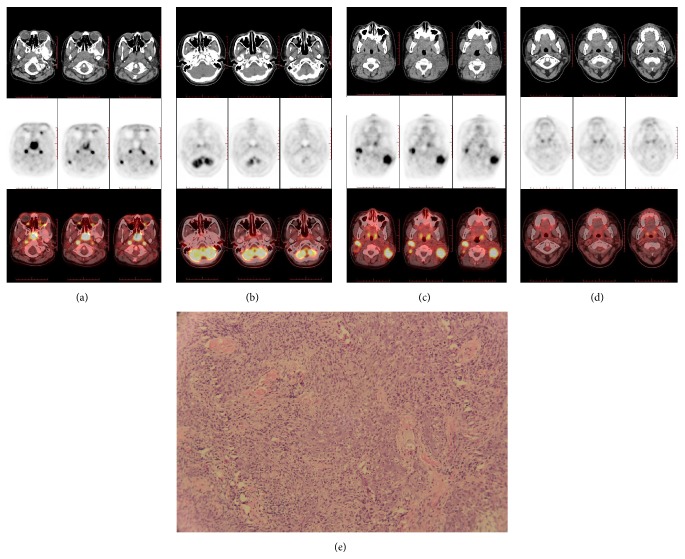
CT (top), PET (middle), and PET/CT (bottom) scans of patient number 1 (a–d). (a) and (c) were for initial staging: pretherapy scans showing diffuse soft-tissue thickening and ^18^F-FDG accumulation in nasopharynx (SUV_max_, 18) (a) and intense uptake in retropharyngeal lymph node and bilateral cervical lymph nodes (SUV_max_, 8.3) (c). (b) and (d) were for follow-up staging: scans after seven cycles of docetaxel-based chemotherapy and nasopharyngeal and cervical radiotherapy showing slight ^18^F-FDG uptake in right nasopharynx (SUV_max_, 4.0) (b) and no uptake in lymph node metastases (d). (e) Photomicrograph of patient number 1 showing poorly differentiated squamous cell carcinoma consistent with NPC (hematoxylin and eosin, ×100).

**Figure 3:**
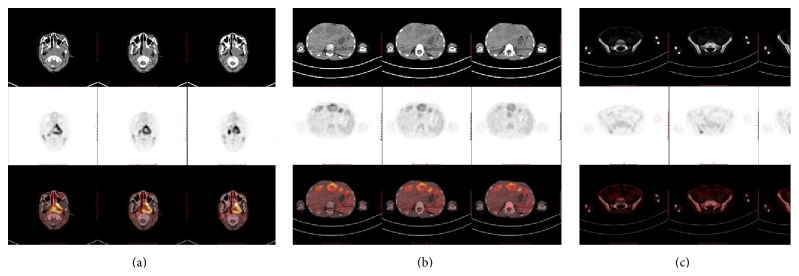
CT (top), PET (middle), and PET/CT (bottom) scans of patient number 3. (a) Intense inhomogeneous ^18^F-FDG uptake in left nasopharyngeal primary mass and asymmetric diffuse soft-tissue thickening in nasopharynx (SUV_max_ 8.2). (b) Multifocal uptake in liver correlating with multiple cystic lesions on CT (SUV_max_ 5.2). (c) ^18^F-FDG uptake in ilium (bone marrow, SUV_max_ 5.0).

The benign increased uptake of ^18^F-FDG in nasopharynx of children under 10 years of age is symmetric and trapezoid (see Figure 1). Nasopharyngeal carcinomas (NPC) (2/17, 12%) were confirmed in 17 suspected malignancies of unknown origin. In 137 established malignancies, 1 (1/137, 0.73%) case had lymphoma involvement. Three patients had pathologically established nasopharyngeal malignancy, two had diffusely and expansively increased FDG uptake, and one had asymmetric uptake. The clinical and imaging characteristics of these three children are listed in Table 2. The SUV_max_ value in patient numbers 1−3 was 18, 11, and 8.2, respectively. Patient number 1 with incidental NPC (SUV_max_, 18; Figure 2) and lymphoma involvement (SUV_max_, 11) had diffusely increased ^18^F-FDG uptake in the nasopharynx (which was not shown). Patient number 3 (Figure 3) had asymmetric ^18^F-FDG uptake in the nasopharynx (SUV_max_, 8.2) and multiple metastases on PET with correlating morphologic lesions on diagnostic CT.

The association between SUV_max_ in the nasopharynx and the incidence of NPC using the 50th, 75th, 90th, and 95th percentiles is presented in Table 3. The risk of nasopharyngeal malignancy increased with SUV_max_: 8.6%, 22%, and 40% in patients with SUV_max_ of at least 7.60, 9.58, and 10.88, respectively.

## 4. Discussion

^18^F-FDG PET/CT is helpful for staging and posttreatment assessment of adult NPC [3–6]. However, in children diffusely increased ^18^F-FDG uptake and thickening of the nasopharynx are common for physiologic and inflammatory reasons. Therefore, it is difficult to tell whether increased nasopharyngeal ^18^F-FDG uptake without a correlating morphologic lesion on diagnostic CT suggests nasopharyngeal malignancy. In this investigation, we retrospectively reviewed nasopharyngeal ^18^F-FDG uptake in 154 children and correlated the SUV_max_ with clinical and pathologic results.

Diffusely increased nasopharyngeal uptake in children may be due to physiologic changes, inflammation, or malignancy. It is not difficult to diagnose asymmetrically increased ^18^F-FDG uptake if there is a corresponding CT abnormality, as illustrated by patient number 3. In our study, uptake having SUV_max_ less than 7.6 in the nasopharynx was considered physiologic. Diffusely increased ^18^F-FDG uptake (SUV_max_ > 9.58) without a correlating morphologic lesion on diagnostic CT may be due to inflammation or nasopharyngeal malignancy. Differentiation between nasopharyngeal inflammation and malignancy using PET/CT is difficult. Our results indicated that, in children under 10 years of age, the typical characteristics of physiologic and inflammatory ^18^F-FDG uptake in nasopharynx are symmetrically trapezoid with the SUV_max_ less than 11. In our study, 7 children had SUV_max_ of more than 9.58 in the nasopharynx due to viral or bacterial infections. However, the higher the nasopharyngeal SUV_max_ (>10.88), the higher the incidence of nasopharyngeal malignancy (2/5, 40%). Nasopharyngeal malignancy in children may be characterized on PET/CT by asymmetric or expansively diffused, increased nasopharyngeal ^18^F-FDG uptake with or without a corresponding CT abnormality with SUV_max_ greater than 11. As shown by patient number 1, nasopharyngeal biopsy may well be indicated in patients with SUV_max_ of more than 11 in the nasopharynx with retropharyngeal space and bilateral cervical lymph node abnormality but no history of malignancy. Meanwhile, carefully diagnosis should be made in patients with SUV_max_ less than 11. Patient number 3 had NPC with SUV_max_ of 8.2 and multiple metastases including liver. The asymmetric ^18^F-FDG uptake and correlating morphologic lesions on diagnostic CT are of great help to make the diagnosis of NPC.

NPC is rare in the pediatric age group. In the study, NPC (2/17, 12%) were confirmed in 17 suspected malignancies of unknown origin. In 137 established malignancies, 1 (1/137,0.73%) case had lymphoma involvement. Children tend to have the poorly differentiated histologic variant of the disease, which is associated with increased locoregional spread and distant metastasis. Splenic metastases have been reported in adult cancers but are unusual in pediatric solid tumors [7]. Bone (67%) and liver (30%) are the most common metastatic sites [8]. PET/CT is valuable for staging of NPC in children. Of the two cases of NPC in our study, one had regional lymphadenopathy and splenic metastases and the other had multiple metastases in bone (bone marrow) and liver that were clearly shown by ^18^F-FDG PET/CT. The rarity of splenic and cystic liver metastases in pediatric NPC has also been highlighted in a case report on a 14-year-old boy [7].

Chemoradiotherapy is the preferred treatment for local disease, whereas chemotherapy is the first choice for children with systemic disease [7]. ^18^F-FDG PET/CT is also helpful in therapeutic monitoring of children with NPC. In patient number 1 the incidental detection of NPC by ^18^F-FDG PET/CT (SUV_max_ 18) was showed in Figures 2(a) and 2(b). PET/CT imaging showed decreased ^18^F-FDG (SUV_max_ 4.0) and negative lymph nodes, which indicated a good response to chemotherapy and local radiotherapy, thus demonstrating the potential usefulness of the technique in metastatic pediatric NPC.

However, there are some limitations of this paper. We excluded malignancy in the other children according to the follow-up PET/CT or CT except for the three patients who had been diagnosed as malignancy. Only three children with nasopharyngeal malignancy were included. Further study will focus on the details about the children with physiologic or inflammatory nasopharyngeal ^18^F-FDG uptake, including the distribution pattern (symmetrical or not) and the correlation of SUVs with the children's age and gender, the soft-tissue thickness of the nasopharynx, and recent history of rhinitis.

## 5. Conclusion

The difference between adenoid hypertrophy and malignancy is asymmetric or diffusely expansive ^18^F-FDG uptake with or without correlating morphologic lesion on diagnostic CT. In children under 10 years of age, the typical characteristics of physiologic and inflammatory ^18^F-FDG uptake in nasopharynx are symmetrically trapezoid. Diffusely increased nasopharyngeal ^18^F-FDG uptake can be considered physiologic if SUV_max_ is less than 7.6 but should be carefully assessed if SUV_max_ is greater than 11 and there is no correlating morphologic lesion on diagnostic CT. The diffusely, expansively increased uptake, and asymmetric uptake in particular, should be considered as malignancy. Further biopsy is especially indicated in patients with retropharyngeal space and bilateral cervical lymph node abnormality but no history of malignancy. ^18^F-FDG PET/CT is a valuable imaging modality in staging and therapeutic assessment of NPC in children.

## Figures and Tables

**Table 1:** Distribution of malignancy in study population.

	Diagnosis	Number of patients
Established malignancy	Osteosarcoma	6
	Lymphoma	7
	Rhabdomyosarcoma	13
	Neuroblastoma	32
	Wilms tumor	21
	Langerhans cell histiocytosis	8
	Brain tumor	27
	Thyroid cancer	3
	Hepatoblastoma	4
	Germ cell tumors	2
	Thymoma	2
	Yolk sac tumor	5
	Medulloblastoma	2
	Primitive neuroectodermal tumors	4
	Lymphoma involvement of nasopharynx	1

Suspected tumors and fever of unknown origin	Nasopharyngeal carcinoma	2
	Others	15

**Table 2:** Clinical features and characteristics of ^18^F-FDG PET/CT in three children with nasopharyngeal malignancy.

Patient number	Age (y)	Sex	Diagnosis at PET scan	PET/CT findings	Results of follow-up
1	10	M	Suspected neurogenic tumors by biopsy of left cervical lymph nodes	Diffusely increased ^18^F-FDG uptake in nasopharynx (SUV_max⁡_, 18), retropharyngeal space, and bilateral cervical lymph nodes; focal uptake in spleen	NPC

2	5	F	Liver multiple metastases of unknown origin	Inhomogeneously increased ^18^F-FDG uptake with irregular thickening in left nasopharynx (SUV_max⁡_, 8.2); multiple liver and bone metastases	NPC

3	6	M	Burkitt lymphoma, tonsils	Diffusely increased ^18^F-FDG uptake in nasopharynx mass (SUV_max⁡_ 11) and bilateral cervical lymph nodes	Lymphoma involvement of nasopharynx

**Table 3:** SUV_max⁡_ and malignancy of nasopharynx in study population.

Median	SUV_max⁡_	Incidence of nasopharyngeal malignancy
50th percentile	5.5	3/75 (4%)
75th percentile	7.6	3/35 (8.6%)
90th percentile	9.58	2/9 (22%)
95th percentile	10.88	2/5 (40%)

## References

[1] Ries L. A. G., Smith M. A., Gurney J. G. (1999). *Cancer Incidence and Survival among Children and Adolescents: United States SEER Program 1975–1995*.

[2] Cheuk D. K., Sabin N. D., Hossain M. (2012). Positron emission tomography-computed tomography for staging and follow-up of pediatric nasopharyngeal carcinoma. *European Journal of Nuclear Medicine and Molecular Imaging*.

[3] Lai V., Khong P. L. (2014). Updates on MR imaging and 18F-FDG PET/CT imaging in nasopharyngeal carcinoma. *Oral Oncology*.

[4] Mohandas A., Marcus C., Kang H., Truong M.-T., Subramaniam R. M. (2014). FDG PET/CT in the management of nasopharyngeal carcinoma. *American Journal of Roentgenology*.

[5] Law A., Peters L. J., Dutu G. (2011). The utility of PET/CT in staging and assessment of treatment response of nasopharyngeal cancer. *Journal of Medical Imaging and Radiation Oncology*.

[6] Gordin A., Golz A., Daitzchman M. (2007). Fluorine-18 fluorodeoxyglucose positron emission tomography/computed tomography imaging in patients with carcinoma of the nasopharynx: diagnostic accuracy and impact on clinical management. *International Journal of Radiation Oncology, Biology, Physics*.

[7] Radhakrishnan V., Thulkar S., Karunanithi S., Tanveer N., Bakhshi S. (2010). Nasopharyngeal carcinoma with splenic and cystic liver metastases in a pediatric patient: 18F-FDG PET-CT findings. *Pediatric Radiology*.

[8] Ayan I., Kaytan E., Ayan N. (2003). Childhood nasopharyngeal carcinoma: from biology to treatment. *The Lancet Oncology*.

